# Update in Antiepidermal Growth Factor Receptor Therapy in the Management of Metastatic Colorectal Cancer

**DOI:** 10.1155/2009/967920

**Published:** 2009-04-09

**Authors:** Herbert H. Loong, Brigette B. Ma, Anthony T. C. Chan

**Affiliations:** ^1^Department of Clinical Oncology, Prince of Wales Hospital, Shatin, New Territories, Hong Kong; ^2^State Key Laboratory in Oncology in South China, Sir YK Pao Centre for Cancer, Department of Clinical Oncology, Hong Kong Cancer Institute and Li Ka Shing Institute of Health Sciences, The Chinese University of Hong Kong, New Territories, Hong Kong

## Abstract

The approval of anti-epidermal growth factor receptor (EGFR) monoclonal antibodies in the treatment of metastatic colorectal cancer (CRC) has expanded the armamentarium against this disease. This paper will review the historical progress and recent clinical developments of anti-EGFR therapies in the treatment of metastatic CRC. Novel strategies of targeting the EGFR pathway to improve efficacy as well as ongoing research in identifying specific molecular predictors of response will be discussed.

## 1. Introduction

A decade ago, the systemic treatment of colorectal cancer (CRC) consisted only of fluoropyrimidine-based chemotherapy administered alone or in combination with either oxaliplatin or irinotecan in an empirical fashion, guided by serial measurements of radiological response. Owing to the remarkable advances in our understanding of the molecular mechanisms of carcinogenesis, target-based therapies are now commonly used as in the treatment of many types of cancer, including CRC. Cetuximab and panitumumab are monoclonal antibody against the epidermal growth factor receptor (EGFR) that has been approved for the treatment of patients with metastatic CRC [1, 2]. The optimal clinical application of anti-EGFR agents in the management of CRC patients and the identification of predictive markers are the main focus of research in recent years. This article will concentrate on the developments and controversies of anti-EGFR therapy in the management of CRC.

## 2. EGFR as a Target in Colorectal Cancer

The concept of manipulation of EGFR in the treatment of epithelial malignancies such as colorectal and lung cancers has actually been envisaged since the mid 1960s [1, 2]. It was during that period that the EGFR protein was first isolated, characterized, and recognized as a potential therapeutic target. Throughout the last 40 years, advances made in basic and clinical research have enhanced our understanding of this target, and many different classes of EGFR inhibitors are now at various stages of clinical development.

EGFR is a 170 kD member of the ErbB receptor tyrosine kinase family of signaling proteins, and its ligands include epidermal growth factor (EGF), transforming growth factor-*α* (TGF-*α*), heparin-binding EGF (HB-EGF), and amphiregulin (AR). Ligand binding, dimerization, and phosphorylation of EGFR lead to activation of downstream proteins, leading to a cascade mediating cell growth and survival [3]. Two different anti-EGFR strategies are currently available in the therapeutic armamentarium: (1) monoclonal antibodies that prevent EGFR ligand binding and (2) tyrosine-kinase inhibitors (TKIs) that block phosphorylation of the intracellular tyrosine kinase component of the EGFR. Both of these strategies dampen signal transduction through some of the downstream pathways such as RAS/RAF/mitogen-activated protein kinase (MAPK) and phosphoinositide 3-kinase (PI3K)-AKT cascades, thus limiting cell growth, proliferation, invasion, angiogenesis, and metastasis [3, 4].

## 3. Anti-EGFR Monoclonal Antibodies

Cetuximab (chimeric IgG1 monoclonal antibody) and panitumumab (fully humanized IgG2 monoclonal antibody) are two anti-EGFR monoclonal antibodies currently approved in the treatment of metastatic CRC. The structural difference between the IgG-1-based cetuximab and IgG-2-based panitumumab is believed to have implications on their mechanisms of action. Preclinical studies have suggested that the cetuximab molecule is able to induce antibody-dependent cell-mediated cytotoxicity (ADCC) [4, 5], where natural killer cells, monocytes, and eosinophils are recruited to lyse the targeted cells (i.e., tumor cells). Being a chimeric antibody, cetuximab is also associated with a slightly higher incidence of hypersensitivity and infusional reactions when compared with fully humanized panitumumab (see Table 1).

### 3.1. Cetuximab

Pharmacokinetics studies have shown that cetuximab's binding affinity for EGFR was shown to be one log higher than its natural ligand and its mechanism of action is thought to be competitive inhibition of ligand binding to EGFR. Cetuximab alone resulted in in vitro and in vivo growth inhibition in multiple tumor types, including CRCs [4, 5]. Cetuximab was able to enhance the antitumor effect of irinotecan, as evident from an experiment on the HT-29 CRC xenograft model, where cetuximab and irinotecan given in combination resulted in a greater degree of tumor growth delay than when either agent was given alone [5]. Furthermore, cetuximab has been shown to overcome acquired resistance against irinotecan in vivo. This was shown as an experiment where the addition of cetuximab resulted in shrinkage of tumor xenografts which were otherwise progressing after previous treatment with irinotecan.

The landmark BOND trial [2] randomized 329 patients with metastatic CRC which were EGFR-positive and refractory to irinotecan, to either cetuximab alone or in combination with irinotecan in a 2 : 1 ratio. EGFR positivity was defined as 1+ staining by immunohistochemistry, and “irinotecan-refractory” status was defined as disease progression on or within 3 months of irinotecan-based therapy. Cross-over was allowed from the monotherapy arm to the combination arm upon disease progression. Objective response rate (ORR) was significantly in favor of the combination arm (22.9% versus 10.8%, *P* = .007). Fifty-six patients who were randomized to cetuximab alone eventually crossed over to the combination arm, while 3.6% and 35.7% of these patients achieved partial response and stable disease, respectively. This study led to the US Food Drug Administration (FDA) approval of cetuximab in patients with irinotecan-refractory meta static CRC. Subsequently, the NCIC-CO.17 study randomized patients who had failed at least 2 lines of prior therapies, to either supportive care or cetuximab alone [6]. In this study where no cross-over was allowed, there was a statistically significant advantage in median overall survival (OS) favoring the cetuximab arm (6.1 months) compared with supportive care (4.6 months). Partial responses occurred in 23 patients (8.0%) in the cetuximab group but none in the group assigned to supportive care alone (*P* < .001).

Cetuximab has also been investigated in the first-line setting. The “CRYSTAL” [7] study is a multicentre phase III trial which randomized more than 1000 patients with metastatic CRC, to either the “FOLFIRI” regimen alone (Irinotecan, infusional 5-fluorouracil and leucovorin in a 2-weekly schedule), or in combination with cetuximab at a weekly schedule. The primary endpoint (progression-free survival (PFS)) was met in the study, where patients randomized to the combination arm had a significantly longer progression-free survival (8.9 months versus 8.0 months; *P* = .036) than the chemotherapy alone arm, but there was no difference in overall survival in the initial intention-to-treat analysis. Response rate was also significantly better in the combination arm (46.9% versus 38.7%; *P* = .005), resulting in a larger number of patients being down staged enough to undergo resection of liver metastases (9.8% versus 4.5%). The “OPUS” study [8] is another first-line randomized phase II study, which randomized 337 chemotherapy-nave patients with metastatic CRC, to either the FOLFOX-4 regimen or in combination with cetuximab in a 1:1 fashion. The overall RR was 45.6% in the combination arm versus 35.7% in FOLFOX-4 alone arm. In the “ACROBAT” study, Tabernero et al. reported on 42 patients who were treated with FOLFOX-4 plus cetuximab, showing a confirmed ORR of 81% [9]. Encouragingly, 10 patients (23%) underwent resection of previously unresectable metastases, 8 of them had liver metastases. The resection with curative intent rate of 23% achieved in this study is therefore comparable with the highest reported for unselected patients.

### 3.2. Panitumumab

The US FDA approval of panitumumab was based on a pivotal multinational phase III study that involved over 400 patients [10]. This study compared panitumumab versus best supportive care (BSC), allowing cross-over from the BSC arm to panitumumab upon disease progression. The median PFS time was 8 weeks for panitumumab and 7.3 weeks for BSC. After a 12-month followup period, response rates were 10% for panitumumab and 0% for BSC (*P* < .0001). The lack of difference in OS (hazard ratio HR 1.00; 95% confidence interval, CI 0.82 to 1.22) maybe attributed to the cross-over design, where 76% of patients in the BSC arm subsequently received panitumumab. As expected with anti-EGFR therapies, skin-related toxicities occurred in 90% of patients in the panitumumab group but no patients had grade 3 or 4 infusional reactions.

## 4. EGFR Tyrosine Kinase Inhibitors

Although not approved for the treatment of CRC, small molecule inhibitors of the EGFR tyrosine kinase (TKI) have been shown to have meaningful activity in different tumor types such as lung and pancreatic cancer. In contrast to EGFR monoclonal antibodies, the site of action of these drugs is intracellular at the ATP-binding site of the EGFR TK domain. Compared with monoclonal antibodies, TKIs may potentially inhibit multiple targets and tend not to induce receptor downregulation.

### 4.1. Gefitinib

Gefitinib is a low-molecular-weight competitive inhibitor of ATP-binding pocket of the EGFR TK domain [11], which is approved for the treatment of nonsmall cell lung cancer. Clinical trials of gefitinib as a single agent in CRC reported no objective tumor responses [12–14] however, a sizable proportion of patients did have disease stabilization. A phase II trial which compared the 250 mg versus a 500 mg daily dosing of gefitinib [15], reported 1 partial response in a patient who received the 500 mg dose. Paired biopsies pre- and posttreatment biopsies performed in 28 patients showed that 84% had no change or increase in the expression level of phosphorylated EGFR, MAP kinase, and Ki67 after treatment. Gefitinib and chemotherapy in combination have also been investigated in several trials, where the response rates seemed to be superior to the historically reported rates of chemotherapy alone [16–18]. However, there were significantly more toxicities particularly with respect to neutropenia and diarrhea with the combination. Studies combining gefitinib with irinotecan also resulted in greater toxicity, with some trials requiring early termination.

### 4.2. Erlotinib

Erlotinib has also been studied in advanced CRC. There have been mixed reports of some clinical activity when used as a single agent. As with gefitinib, erlotinib produced a higher response rate when combined with chemotherapy. High incidence of toxicity was noted when given in combination with systemic chemotherapy, especially in two trials where erlotinib was given in combination with FOLFOX and bevacizumab [19, 20], with the latter trial having prematurely closed due to toxicity.

## 5. Incidence and Implications of Skin Rash

Anti-EGFR monoclonal antibodies and EGFR TKIs are associated with a distinctive skin rash. The rash is characterized histologically as a neutrophilic infiltrate in perifollicular areas within the basal layer of the skin, which is different from that seen in typical acne. Skin toxicity is generally observed within 2 to 3 weeks after the start of treatment and gradually resolves in most patients, even when anti-EGFR treatment is continued. In the BOND study, the most frequently observed adverse event to cetuximab was the skin rash, and in the panitumumab study, Hecht et al. [21] reported a 95% incidence of acneiform skin rash of any grade. Grade 3 rash occurred in 3% of patients and none experienced grade 4 skin toxicities. An association between the severity of acneiform rash and efficacy to cetuximab has been well described. Retrospective analysis of the BOND data showed a clear association between higher grades of skin reaction with RR and time to progression (TTP) [2]. This association is also seen with panitumumab [10] and seems to hold true in the treatment of tumors of other sites with this class of agents.

The “EVEREST” study [22] was a phase I/II dose-escalation study, where patients who were receiving cetuximab were randomized at 22 days, to either standard dose of weekly (250 mg/m2/week) cetuximab or an escalating dose of cetuximab until the development of grade 3 toxicity, with a maximum ceiling dose of 500 mg/m2/week. Skin and tumor biopsies were obtained. Preliminary report suggested that the PFS in standard-dose arm was 3.9 months and 4.8 months in the dose-escalated arm. Dose-related increases in pharmacokinetic parameters (e.g., Cmax, AUC) were observed in the escalated arm. The authors concluded that cetuximab dose escalation up to 500 mg/m2/w may improve ORR in patients who experienced no or slight skin reactions on standard-dose cetuximab.

## 6. Combining Targeted Therapies in Colorectal Cancer

Combinations of multiple targeted agents with or without the additional of chemotherapy have also been investigated. The BOND-2 trial [23] randomized irinotecan-refractory patients to either 2 drugs (bevacizumab and cetuximab) or 3 drugs (bevacizumab, cetuximab and irinotecan). The results were encouraging, with the 3-drug arm resulting in a better TTP and ORR than the 2-drug arm. This study also showed for the first time that monoclonal antibodies in combination could induce an ORR of 20% in the absence of chemotherapy. Nonstatistical comparison with result of the BOND-1 study suggested that bevacizumab may enhance the effects of irinotecan-cetuximab combination, with an ORR of 37% as compared to 23% reported for the cetuximab/irinotecan arm [2, 23] (see Table 2). Subsequent to the BOND-2 study, 3 other randomized trials have been reported on the feasibility of combining monoclonal antibodies in the treatment of CRC. The PACCE study [24] randomized 824 treatment-nave patients with metastatic CRC to oxaliplatin-based chemotherapy plus bevacizumab, with or without panitumumab, and 230 patients to irinotecan-based chemotherapy plus bevacizumab with or without panitumumab. The study was terminated at a preplanned interim analysis after 231 events were reported in patients who received oxaliplatin-based therapy, where a statistically significant increase in PFS was reported in the arm without panitumumab. In contrast, the Dutch CAIRO-2 trial [25] has a similar study design to the PACCE trial and involved over 700 patients. Patients received capecitabine, oxaliplatin, and bevacizumab with or without additional cetuximab. As expected, preliminary result reported a significantly higher incidence of grade 3-4 skin rash in the cetuximab-containing arm, without a statistically significant difference in mortality in either arm. The CALGB/SWOG 80405 intergroup trial was a 3-arm study, which randomized patients with metastatic CRC to chemotherapy and cetuximab, chemotherapy and bevacizumab, or chemotherapy plus cetuximab and bevacizumab. This study has been suspended following a decision by the CALGB Data and Safety Monitoring Board as from June 2008, pending a protocol revision in view of the data on KRAS mutation (see below) [26]. The reason why the PACCE study failed to demonstrate a survival advantage in the panitumumab-bevacizumab arm remains unclear. It is possible that the dose of the antibodies was inappropriate leading to excessive toxicity and reduced efficacy. Patient selection has also proven critical in the optimal use of anti-EGFR antibodies as the use of EGFR staining to predict response and outcome has been severely challenged. Thus, a combination approach of targeted agents in metastatic CRC remains controversial and it is the consensus among gastrointestinal oncologists that such an approach remains experimental.

## 7. EGFR Expression in Colorectal Cancer

EGFR as determined by immunohistochemical (IHC) methods was the first biomarker investigated as a potential predictor of response. It is overexpressed in over 80% of colorectal cancers [27]. However, EGFR expression as measured by immunohistochemistry does not predict clinical benefit [28, 29]. Initial observations in a small retrospective series by Lenz et al. [30] noted that more than 20% of EGFR-negative patients developed major objective responses. An extensive retrospective analysis was reported in 2005 by Chung et al. [29]. They reported a response rate of 25% in EGFR-negative patients (4 out of 16) given cetuximab and irinotecan. This was comparable and indistinguishable from the response rate of 23% seen in two separate clinical trials with EGFR-positive patients [1, 31]. Given this data, immunohistochemical (IHC) demonstration of EGFR expression is no longer required before starting cetuximab therapy in practice. Similarly, in the phase II trial, the response to treatment with panitumumab in patients with metastatic CRC was similar, irrespective of the level of EGFR protein expression assessed by IHC analysis [32].

## 8. Potential Predictors of Response to Anti-EGFR Therapy

### 8.1. Activating EGFR Gene Mutations

There has been much interest in determining whether EGFR gene mutations may play a role in affecting response to cetuximab or panitumumab. Previous research in nonsmall cell lung cancer [33] has shown that EGFR TK mutations predict benefit from EGFR TKIs. However, several retrospective studies on tumor biopsies and cell lines found that EGFR gene mutations in CRC are extremely rare [34, 35]. No significantly different gene mutations were found between responders and nonresponders to treatment.

### 8.2. EGFR Gene Amplification

It has been hypothesized that DNA is a more stable molecule than a protein and thus EGFR gene copy number maybe more accurate reflection of the EGFR status than IHC expression [36]. In a cohort of patients treated with cetuximab or panitumumab, EGFR gene amplification with increased copy number has been shown to significantly correlate with objective response to treatment [36]. This raises the possibility that patients with high EGFR copy number may be more likely to respond to treatment with anti-EGFR therapies.

### 8.3. KRAS Gene Mutation

KRAS is a guanosine triphosphate (GTP)-binding protein that acts as a critical on-off switch in cellular growth and survival pathways. It plays a key role in the RAS/MAPK signally pathway located downstream of many growth factor receptors, including EGFR, and involved in carcinogenesis. Mutations of KRAS that result in the constitutive activation of MAPK pathway downstream occurs in about 40% of colorectal cancers [37]. A retrospective analysis reported by Lièvre et al. [38] analyzed tumor samples from 30 patients treated with cetuximab. A KRAS mutation was found in 13 tumors (43%) and was significantly associated with absence of response to cetuximab. None of the patients with response to cetuximab harbored a KRAS mutation. The overall survival of patients without KRAS mutation was significantly higher compared with those patients with a mutated tumor (*P* = .016; median survival 16.3 versus 6.9 months). An increase in EGFR copy number was also significantly associated with objective tumor response.

It has been hypothesized that irrespective of the level of EGFR expression, the presence of a KRAS mutation is associated with a constitutive activation of the RAS/MAPK pathway, leading to cell proliferation which cannot be significantly inhibited by cetuximab. KRAS mutations have also been implicated in resistance against EGFR TKIs in lung adenocarcinomas [39].

Large scale retrospective reviews retrieved archived tumor tissue from prior cetuximab and panitumumab trials. KRAS mutation analysis was performed tested in tumor samples collected from over 1000 participants of the “CRYSTAL” [8], “OPUS” [40], NCIC-CO.17 trial [41] and panitumumab trials [42]. Beneficial effects of anti-EGFR antibodies were limited to a subgroup of patients with wild-type KRAS tumors. This has led to the recommendation that all patients with advanced CRC who are being considered for cetuximab or panitumumab should undergo KRAS testing, and if the cancer bears a mutated KRAS gene, they should not receive an antibody that targets EGFR.

## 9. Conclusion

Throughout the last decade, significant advancement in our understanding of the molecular mechanisms of metastatic CRC has been made. Anti-EGFR monoclonal antibodies approved for use in the metastatic setting have broadened the therapeutic armamentarium in the treatment of metastatic CRC. The most effective sequence and combinations of anti-EGFR therapy with chemotherapy and, or bevacizumab in order to achieve cytotoxic potentiation with limited toxicity need to be addressed. Advances made in the identification of predictive biomarkers such as KRAS mutations allow us to select distinct groups of patients who are most likely to benefit from cetuximab therapy.

## Figures and Tables

**Table 1:** Comparison between cetuximab and panitumumab.

	Cetuximab	Panitumumab
Structure	Chimeric IgG-1,	Fully humanized: IgG-2
	30% murine	
Hypersensitivity reaction	3%	1%
Half life	5 days	7.5 days
Treatment schedule	1-2 weekly	2-weekly
Antibody-dependent cell	Yes	No ADCC reported
mediated cytotoxicity	Fc Domain	
(ADCC)	of IgG-1	

**Table 2:** Nonstatistical comparison of the results of the BOND-2 [23] and BOND-1 study [1].

BOND2	BOND2		BOND1	BOND1
Cetuximab, irinotecan, and bevacizumab	Cetuximab and bevacizumab		Cetuximab and irinotecan	Cetuximab alone
No. of Patients	43	40	218	111
Prior treatment with oxaliplatin (%)	87	89	62	64
Response rates (%)	37	20	23	11
Time to progression (months)	7.3	4.9	4.1	1.5
Median overall survival (months)	14.5	11.4	8.6	6.9
